# Vitamin D release across abdominal adipose tissue in lean and obese men: The effect of ß‐adrenergic stimulation

**DOI:** 10.14814/phy2.14308

**Published:** 2019-12-23

**Authors:** Adriyan Pramono, Johan W. E. Jocken, Gijs H. Goossens, Ellen E. Blaak

**Affiliations:** ^1^ Department of Human Biology NUTRIM School of Nutrition and Translational Research in Metabolism Maastricht University Maastricht The Netherlands; ^2^ Department of Nutrition Science Faculty of Medicine Universitas Diponegoro Indonesia

**Keywords:** ß‐adrenergic stimulation, arterio‐venous, lipolysis, obesity, vitamin D

## Abstract

Obesity is characterized by a blunted lipolytic response in abdominal subcutaneous adipose tissue (SAT) and low circulating vitamin D levels. Here we investigated whether an impaired SAT lipolytic response coincides with an impaired SAT vitamin D release in eight lean and six obese men. 25‐hydroxyvitamin D_3_ [25(OH)D_3_] and 1,25‐dihydroxyvitamin D_3_ [1,25(OH)_2_D_3_] fluxes across SAT were measured using arterio‐venous blood sampling in combination with AT blood flow measurements after an overnight fast and during 1‐hr intravenous infusion of the non‐selective ß‐adrenergic agonist isoprenaline (20 ng·kg FFM^−1^·min^−1^). 1,25(OH)_2_D_3_ was released across abdominal SAT during isoprenaline infusion in lean [−0.01 (−0.04 to 0.00) pmol*100 g tissue^−1^*min^−1^, *p* = .017 vs. zero flux], but not in obese men [0.01 (0.00 to 0.02) pmol*100 g tissue^−1^*min^−1^, *p* = .116 vs. zero flux], and accompanied by an impaired isoprenaline‐induced lipolytic response in abdominal SAT of obese versus lean men. Isoprenaline had no significant effects on net 25(OH)D_3_ release across abdominal SAT and plasma vitamin D metabolites in lean and obese men. To conclude, a blunted isoprenaline‐mediated lipolysis is accompanied by reduced release of 1,25(OH)_2_D_3_ vitamin D across abdominal SAT in obesity.

## Graphical abstract

Low circulating levels of vitamin D are often linked with obesity. Sequestration of this fat‐soluble vitamin has been proposed as a putative explanation for the low circulating vitamin D‐levels in obese. In the present manuscript we show that there may be a reduced release of the active metabolite of vitamin D from abdominal subcutaneous adipose tissue in obese individuals, using a tissue balance technique.

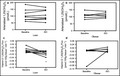

## 1 INTRODUCTION

Obesity is associated with adipose tissue (AT) dysfunction, which is characterized by adipocyte hypertrophy, AT inflammation, and impaired lipid metabolism, thereby contributing to insulin resistance (Stinkens, Goossens, Jocken, & Blaak, 2015). Noteworthy, obesity is often characterized by low circulating vitamin D levels (Vanlint, 2013). In line, studies have described an inverse association between body mass index (BMI) and circulating concentration of the inactive vitamin D metabolite 25‐hydroxyvitamin D_3_ (25(OH)D_3_) (Pourshahidi, 2015). Obesity‐associated insulin resistance is often accompanied by dysfunctional adipose tissue (AT), which might also contribute to increased vitamin D sequestration and an impaired vitamin D release in human obesity (Pramono, Jocken, & Blaak, 2019).

Uptake and sequestration of vitamin D in the expanded obese AT mass may partly contribute to the relatively low vitamin D concentrations in the circulation in obesity (Wortsman, Matsuoka, Chen, Lu, & Holick, 2000), although the underlying mechanism is not yet clearly understood. Furthermore, evidence in rodents suggests that vitamin D may also be released from adipose tissue into the circulation (Rosenstreich, Rich, & Volwiler, 1971).

Obesity is characterized by increased lipid storage in the form of triacylglycerol (TAG), mainly in adipose tissue. Catecholamine stimulation leads to an increase in adipose tissue lipolysis, thereby resulting in the hydrolysis of TAG stored in lipid droplets (Stinkens et al., 2015). Since vitamin D is a lipophilic vitamin that has been postulated to accumulate in adipose tissue, deliberation of TAG may coincide with release/mobilization of vitamin D metabolites from adipose tissue (Hengist et al., 2019). In line with our hypothesis, it has shown that adipose tissue derived from obese individuals releases less vitamin D ex vivo when stimulated with adrenaline compared to lean subjects (Di Nisio et al., 2017). In the latter study, impaired mobilization of vitamin D coincided with blunted catecholamine‐induced lipolytic response, determined by glycerol release into the medium. Nevertheless, other mechanisms like competition between some free fatty acids and vitamin D for binding sites to vitamin D‐binding protein (DBP) (Bouillon, Xiang, Convents, & Baelen, 1992) or a role of adipose tissue blood flow (ATBF) in relation to both lipolysis and vitamin D release may possibly be involved.

Therefore, it is tempting to speculate that low circulating vitamin D levels in human obesity might be due to an increased uptake and/or a blunted release of vitamin D across abdominal subcutaneous AT (SAT), which may occur concurrently with the often observed blunted catecholamine‐mediated lipolysis (Jocken et al., 2008). However, it remains to be determined whether ß‐adrenergic stimulation induces vitamin D 25(OH)D_3_ [inactive metabolite] as well as 1,25(OH)_2_D_3_ [active metabolite] release across human SAT in vivo.

In this study, using arterio‐venous methodology, we investigated (1) the effect of ß‐adrenergic stimulation on net release of vitamin D 25(OH)D_3_ [inactive] and 1.25(OH)_2_D_3_ [active metabolite] across abdominal SAT in lean and obese men, and (2) whether an impaired release of vitamin D across obese abdominal SAT is accompanied by a blunted lipolytic response in obese men.

## 2 METHODS

### 2.1 Study participants

The participants of this study were a subset of previous study (Jocken et al., 2008). Eight lean (BMI < 25 kg/m^2^) and six obese (BMI > 30 kg kg/m^2^) men were included in this analysis. Inclusion criteria for both groups were that participants had to be weight‐stable (weight change < 3.0 kg) for at least 3 months prior to the study, were in good health as assessed by medical history, were free of any medication and spent not more than 3 h of organized sports activities a week. Exclusion criteria were smoking, cardiovascular disease, type 2 diabetes mellitus, liver, or kidney malfunction, use of medication known to affect body weight and glucose metabolism, or untreated hypertension. The Medical Ethical Committee of Maastricht University (MEC‐03‐179) approved the study which was performed according to the procedures set by the latest version of the Declaration of Helsinki, and a written informed consent was obtained from all participants.

### 2.2 Study design

In this study, participants were allowed to perform only light‐intensity physical activity (for examples: walking slowly (in the office), sitting in front of computer/TV, and no sports activities) 2–3 prior to the test day. All participants were asked to refrain from drinking alcohol and to perform no strenuous exercise for 24 hr before the study. Participants came to the university and underwent arterio‐venous (A‐V) blood sampling across abdominal SAT after an overnight fast and after 1 hr intravenous infusion of the nonselective ß‐adrenergic agonist isoprenaline (20 ng (kg FFM)^−1^ min^−1^), as described previously (Jocken et al., 2008). Circulating concentrations and fluxes across abdominal SAT of glycerol and vitamin D were measured following a 3 hr primed (3 µmol·kg^−1^) constant infusion of [^2^H_5_]glycerol (0.2 µmol·kg^−1^). Blood samples were taken simultaneously from the arterialized venous blood was sampled from a superficial dorsal hand vein and adipose vein at three baseline time points (*t*90, *t*105 and *t*120 min) and at three time points during the last 30 min of isoprenaline infusion (*t*150, *t*165, and *t*180 min). Adipose tissue blood flow (ATBF) was monitored continuously using the ^133^Xe wash‐out technique. Lean and obese subjects were studied throughout the year in random order, thus the comparison between groups was not confounded by seasonal variation.

### 2.3 Laboratory analysis

Blood samples were transferred into ice‐chilled polypropylene tubes and were centrifuged (1,000*g*, 4°C, 10 min). Plasma was immediately frozen in liquid nitrogen and safely stored at −80°C until analyses. It has been previously demonstrated that when immediately frozen (at −80°C), vitamin D metabolites are stable for many years (Agborsangaya* et al., 2009; Colak, Toprak, Dogan, & Ustuner, 2013; El‐Khoury & Wang, 2012; Müller, Stokes, Lammert, & Volmer, 2016; Ocké et al., 1995). Vitamin D 25(OH)D_3_ and 1,25(OH)_2_D_3_ levels were measured in arterialized and venous plasma samples. Plasma samples at time‐points t90, t105 and t120 min (steady state for lipolysis at baseline) and t150, t165, and t180 min (steady state for lipolysis during ISO) were pooled because of a lack of sample material at the same time point for all subjects. There was a steady state at baseline as well as during ISO so that pooling was justified. Vitamin D metabolites were analyzed using liquid‐chromatography tandem‐mass spectrophotometry (LC–MS/MS) (Ter Horst et al., 2016). Stable isotope enrichment of glycerol was measured using GC–MS as described previously (Jocken et al., 2008).

### 2.4 Calculations

Net vitamin D 25(OH)D_3_ and 1,25(OH)_2_D_3_ fluxes across SAT were calculated by multiplying arterial‐venous (A‐V) concentration differences by adipose tissue plasma flow, as described in other contexts (Goossens et al., 2008; Jocken et al., 2008). Tissue blood flow during baseline is an average of time‐points t90, t105, t120 min and during ISO is an averaged of t150, t165, and t180 min. Plasma flow was calculated as tissue blood flow multiplied by (1 − hematocrit/100), with hematocrit expressed as a fraction. Positive fluxes indicate net uptake from the circulation, whereas negative fluxes indicate net tissue release into the circulation. The SAT total glycerol uptake was calculated as described previously according to the steady state Steele's equation. Abdominal SAT total glycerol release was calculated by subtracting abdominal SAT net glycerol flux to abdominal SAT total glycerol uptake (Jocken et al., 2008).

### 2.5 Statistical analysis

Subjects characteristics were normally distributed, data are presented as mean ± standard deviation (*SD*), and differences between lean and obese were tested using Student's unpaired *t* test. Since vitamin D metabolites, net fluxes, and adipose tissue blood flow were not normally distributed, a non‐parametric Mann–Whitney test was used for group comparisons. The effects of ß‐adrenergic stimulation within groups were tested using the Wilcoxon signed‐rank test, and data presented as median (range). Kruskal–Wallis test was performed in order to analyze the difference between groups. A Spearman correlation was performed to analyse the relationships between vitamin D fluxes, glycerol, NEFA, and circulating vitamin D levels. Statistical calculations were performed with SPSS for Macintosh (version 21.0; SPSS).

## 3 RESULTS

### 3.1 Subject characteristics

Table 1 shows that lean and obese subjects had a comparable age. By definition, BMI, body fat percentage, body fat mass as well as homeostasis model assessment for insulin resistance (HOMA‐IR) were significantly higher in obese compared with lean participants (all *p* < .01).

**Table 1:** Characteristics of participants

Characteristics	Lean (*N* = 8)	Obese (*N* = 6)
Age (years)	50 ± 9	53 ± 9
BMI (kg/m^2^)	23.7 ± 1.3	32.3 ± 2.2**
Waist (cm)	88.9 ± 3.1	110.2 ± 7.3**
WHR	0.9 ± 0.03	1.0 ± 0.03*
BF (%)	21.5 ± 3.0	31.8 ± 1.6**
FM (kg)	16.2 ± 2.0	31.4 ± 4.5**
HOMA‐IR	1.8 ± 0.7	3.6 ± 1.0*

*p<.01; ** *p*<.001, values are mean ± *SD*.

Abbreviations: BF, body fat; BMI, body mass index; FM, fat mass; HOMA‐IR, homeostatic model assessment for insulin resistance; WHR, Waist to hip ratio.

### 3.2 Adipose tissue blood flow (ATBF)

Adipose tissue blood flow (ATBF) was comparable between lean and obese individuals at baseline (*p* = .108). As expected, Isoprenaline significantly increased ATBF both in lean [1.8 (1.3–2.9) vs. 4.8 (2.6–11.1) ml (100 g tissue)^−1^min^−1^; *p* = .01) and obese [1.3 (1.2–2.4) vs. 3.2 (2.1–6.2) ml (100 g tissue)^−1^min^−1^; *p* = .03] men. Importantly, this increase was not different between groups (*p* = .245).

### 3.3 Systemic (arterialized) concentrations and net vitamin D release across abdominal SAT

#### 3.3.1 Vitamin D 25(OH)D_3_

Plasma arterialized 25(OH)D_3_ did not differ between lean and obese individuals at baseline (56.1 (16.0–84.5) vs. 49.6 (33.5–63.4) nmol/L, respectively, *p* = .852). Additionally, no net 25(OH)D_3_ release across abdominal SAT was observed at baseline in both lean [0.02 (−5.2 to 5.0) pmol*100 g tissue^−1^*min^−1^, *p* = 1.000 vs. zero flux] and obese [0.70 (−4.9 to 1.7) pmol*100 g tissue^−1^*min^−1^, *p* = .917 vs. zero flux].

**Figure 1:**
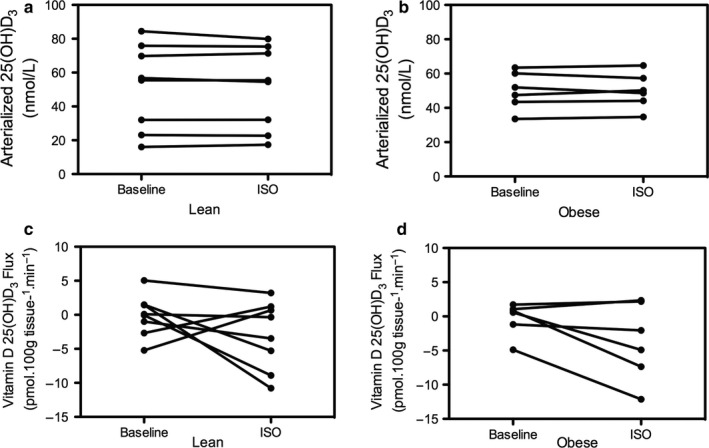
Panel a and b depict plasma (arterialized) Vitamin D 25(OH)D_3_ concentration at baseline and following ISO in lean (*n* = 8) and obese (*n* = 6). Net vitamin D 25(OH)D_3_ release (flux) across abdominal SAT in lean (Panel c) and obese (Panel d)

Following ß‐adrenergic stimulation plasma (arterialized) 25(OH)D_3_ level was not significantly increased in lean [baseline: 56.1 (16.0–84.5) vs. isoprenaline 55.0 (17.4–80.0), *p* = .499] (Figure 1a) or obese men [baseline: 49.6 (33.5–63.4) vs. 49.3 (34.6–64.6), *p* = .917] (Figure 1b). In line, ß‐adrenergic stimulation did not significantly induce net 25(OH)D_3_ release across abdominal SAT in lean [−1.9 (−10.8–3.2) pmol*100 g tissue^−1^*min^−1^, *p* = .208 vs. zero flux] (Figure 1c) and obese [−3.4 (−12.1 to 2.4) pmol*100 g tissue^−1^*min^−1^, *p* = .249 vs. zero flux] (Figure 1d).

#### 3.3.2 Vitamin D 1,25(OH)_2_D_3_

Plasma arterialized 1,25(OH)_2_D_3_ did not differ between lean and obese individuals at baseline (96.6 (60.7–185.7) vs. 108.2 (80.5–147.7) pmol/L, respectively, *p* = .755). Furthermore, no net 1,25(OH)_2_D_3_ release across SAT was observed under baseline conditions in lean [0.00 (−0.01 to 0.03) pmol*100 g tissue^−1^*min^−1^, *p* = .889 vs. zero flux] and obese men [0.00 (−0.09 to 0.00) pmol*100 g tissue^−1^*min^−1^, *p* = .345 vs. zero flux].

**Figure 2:**
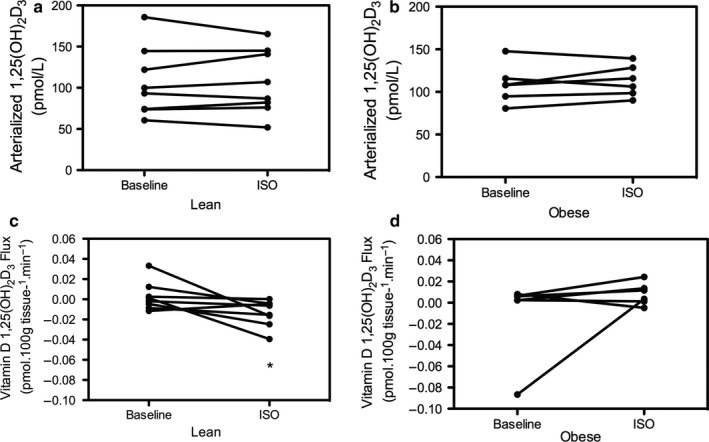
Plasma (arterialized) vitamin D 1,25(OH)_2_D_3_ concentration at baseline and following ISO in lean (Panel a) versus obese (Panel b). Net vitamin D 1,25(OH)_2_D_3_ release (flux) across abdominal SAT in lean (Panel c) and obese (Panel d). (*) *p* < .05 versus zero flux

Following ß‐adrenergic stimulation plasma (arterialized) 1,25(OH)_2_D_3_ was not significantly increased in lean [baseline: 96.6 (60.7–185.7) vs. isoprenaline 97.0 (51.9–165.2), *p* = .889] (Figure 2a) or obese men [baseline: 108.2 (80.5–147.7) vs. 111.1 (90.1–139.3), *p* = .528] (Figure 2b). However, the ISO induced change in 1,25(OH)_2_D_3_ fluxes was significantly different between groups (*p* = .007). An increased net 1,25(OH)_2_D_3_ release across abdominal SAT was observed in lean [ISO: −0.01 (−0.04 to 0.00), *p* = .017 vs. zero flux] (Figure 2c), but not in obese men [ISO: 0.01 (0.00–0.02), *p* = .116 vs. zero flux] (Figure 2d).

### 3.4 Relationship between AT lipolysis and vitamin D (arterialized) concentrations

**Figure 3:**
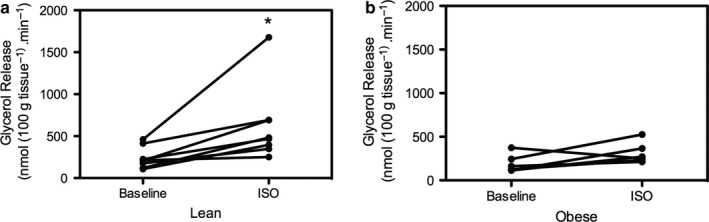
Total glycerol release across abdominal SAT at baseline and during ß‐adrenergic stimulation in lean (*n* = 8) versus obese (*n* = 6). Total glycerol release following ß‐adrenergic stimulation was significantly higher in lean (Panel a) versus obese (Panel b). (*) *p* < .05

Next, we investigated whether the observed vitamin D release across SAT was associated with changes in local lipolytic responses and systemic vitamin D concentration. As reported previously, a blunted ß‐adrenergic mediated increase in total glycerol release across abdominal SAT was observed in obese [glycerol baseline vs. ISO obese: 143.9 (114.4–373.5) vs. 260.5 (213.2–526.1) nmol·100 g tissue^−1^·min^−1^; (*p* = .11, Figure 3b)] compared to lean men [glycerol baseline vs. ISO lean: 209.6 (170.8–460.2) vs. 474.8 (250.8–1678.2) nmol·100 g tissue^−1^·min^−1^); (*p* = .01, Figure 3a)]. However, in this study, net vitamin D 1,25(OH)_2_D_3_ release (flux) was not correlated with plasma (arterialized) glycerol levels during ß‐adrenergic stimulation nor with circulating (arterialized) non‐esterified fatty acid (NEFA) and 1,25(OH)_2_D_3_ levels. (*p* > .05 for all, Supplemental Table S1).

## 4 DISCUSSION

Obesity is often associated with vitamin D deficiency, which has been suggested to relate to insulin resistance and an impaired metabolic health (Pramono et al., 2019). Recent studies in humans have demonstrated that vitamin D (including its metabolites) accumulates and might be metabolised in adipose tissue (Carrelli et al., 2017; Didriksen, Burild, Jakobsen, Fuskevåg, & Jorde, 2015; Wamberg et al., 2013). It has been shown that at least 35% of circulating vitamin D is likely sequestered in human subcutaneous adipose tissue (Heaney, Horst, Cullen, Armas, & a. G., 2009). It was recently demonstrated that beside vitamin D uptake by adipose tissue there also can be significant release both in mice as well as human adipocytes (Bonnet et al., 2018). The latter findings suggest that beside an increased sequestration of vitamin D in AT, also an impaired release (Di Nisio et al., 2017), may contribute to the accumulation of vitamin D in adipose tissue and to the often observed reduced circulating vitamin D concentrations in obesity.

In this study, we investigated in vivo fluxes of 25(OH)D_3_ and 1,25(OH)_2_D_3_ across abdominal SAT for the first time after an overnight fast and during short‐term ß‐adrenergic stimulation in lean and obese men using the arterio‐venous balance methodology. We observed that net vitamin D 1,25(OH)_2_D_3_ release across abdominal SAT during ß‐adrenergic stimulation was significantly higher in lean as compared to obese men, suggesting a blunted vitamin D 1,25(OH)_2_D_3_ release across abdominal SAT in obese men in vivo. In contrast, no significant 25(OH)D_3_ release across SAT was observed in lean or obese men following an overnight fast or during ß‐adrenergic stimulation. The latter findings may be in contrast with recent ex vivo data, which showed a more pronounced reduction in 25(OH)D_3_ content in SAT derived adipocytes in lean compared to obese donors following adrenaline stimulation, possibly pointing toward a blunted 25(OH)D_3_ release in obese SAT (Di Nisio et al., 2017). Unfortunately, in that study only measured vitamin D 25(OH)D_3_ tissue content and not 1,25(OH)_2_D_3_ or release in the medium. Using ex vivo cultures, the adipocytes are withdrawn from their natural local hormonal microenvironment, which may also partly explain the differences with the present in vivo findings.

The reason for the blunted SAT release of 1,25(OH)_2_D_3_ and not 25(OH)D_3_ in obese individuals remains to be determined. Factors like variation in ATBF theoretically resulting in a differential supply of vitamin D carriers to SAT or differences in circulating NEFA, interfering with the binding of vitamin D to its carrier could possibly explain the difference in vitamin D release. However, the ISO‐induced increase in blood flow as well as circulating NEFA were not different between groups. Moreover the above mechanisms would not explain why the differential release is only observed for 1,25(OH)_2_D_3_ and not for 25(OH)D_3._ Vitamin D 1,25(OH)_2_D_3_ is a ligand for vitamin D receptor (VDR), and it has been shown that vitamin D receptor (VDR) expression is increased in SAT of individuals with obesity (Jonas et al., 2019). From the latter, it could be speculated that vitamin D 1,25(OH)_2_D_3_ binds to a higher extent to VDR within SAT in individuals with obesity, resulting in less spillover of vitamin D 1,25(OH)_2_D_3_ in the circulation in obese individuals but not in lean, which still needs further investigation.

Of interest, the observed blunted release of 1,25(OH)_2_D_3_ across abdominal SAT that we found in the present study was accompanied by (but not correlated with) a blunted glycerol release in SAT of obese men. The latter finding as well as the fact that the blunted lipolysis only coincides with a blunted release of 1,25(OH)_2_D_3_ and not 25(OH)D_3_ does not support the idea that it is in the obese individuals observed impaired TAG hydrolysis that drives a blunted release of vitamin D metabolites. The exact relationship between the impaired ISO‐induced lipolysis and 1,25(OH)_2_D_3_ release and to what extent they are co‐regulated remains to be determined.

Furthermore, although net 1,25(OH)_2_D_3_ release was observed during acute ß‐adrenergic stimulation, no changes in plasma vitamin D 1,25(OH)_2_D_3_ (arterialized) concentration were observed. It is likely that following this short‐term (1 hr) ISO infusion, the contribution of 1,25(OH)_2_D_3_ release per unit adipose tissue may be relatively too small to induce significant changes in circulating vitamin D concentrations. We have estimated this based on several assumptions for the amount of total body water (72% of fat free mass and 10% of fat mass is water) and extra cellular water (38% of total body water) (McArdle, 2010) and we took into account the half‐life of 1,25(OH)_2_D_3_ (Lips, 2007). Based on these assumptions, the estimated percentage contribution of total vitamin D 1,25(OH)_2_D_3_ release across adipose tissue to plasma concentrations during isoprenaline stimulation ranged between 0% and 4%. This relatively small contribution might partly explain why no significant increase in plasma vitamin D 1,25(OH)D_3_ after ß‐adrenergic stimulation was observed in the present study.

We have measured vitamin D 25(OH)D_3_ and 1,25(OH)_2_D_3_ metabolites based on their proposed importance in human metabolism and health (DeLuca, 2014). However, there are several other vitamin D metabolites such as 24,25‐dihydroxyvitamin D [24,25(OH)_2_D_3_] (Bosworth et al., 2012) and 3‐epi‐25(OH)D (Lensmeyer, Poquette, Wiebe, & Binkley, 2012). The role of the latter vitamin D metabolite (Zheng et al., 2018) in human is currently unknown and warrants investigation. A limitation of the study is that we were, unfortunately, unable to measure adipose tissue vitamin D content, which would be interesting to take into account in future studies.

Whether long‐term interventions that activate SAT lipolysis and vitamin D release (e.g., weight loss (Gangloff et al., 2015) and exercise (Hengist et al., 2019)) might affect circulating vitamin D concentrations needs to be investigated in more detail. In conclusion, our unique in vivo data show that ß‐adrenergic stimulation induces release of active vitamin D metabolite across abdominal SAT. In thisstudy, a blunted catecholamine‐mediated lipolysis was accompanied by a decreased 1,25(OH)_2_D_3_ (active metabolite) release across abdominal SAT in obese men. Future studies are warranted to elucidate to what extent this blunted vitamin D release may affect circulating vitamin D concentrations in obese humans.

## CONFLICT OF INTEREST

The authors declare that they have no conflict of interest.

## AUTHOR CONTRIBUTIONS

A.P. and J.W.E.J. designed the study and collected data. A.P. analyzed data. Data interpretation was performed by A.P., J.W.E.J., G.H.G., and E.E.B. The manuscript was written by A.P. and was revised by J.W.E.J., G.H.G., and E.E.B. All authors reviewed and approved the final manuscript.
