# Supplementary material for: Vitamin D release across abdominal adipose tissue in lean and obese men: The effect of ß‐adrenergic stimulation
Source: Physiol Rep. 2019 Dec 23;7(24):e14308. doi: 10.14814/phy2.14308 (PMC6928243; doi:10.14814/phy2.14308)
Supplement: Supplementary file 1 [file PHY2-7-e14308-s001.docx]

**Supplemental Table S1. Spearman correlation coefficient between net vitamin D release and arterialized glycerol, arterialized NEFA levels, arterialized vitamin D 1,25(OH)_2_D_3_ during ISO concentrations.**

|  | **Arterialized Glycerol** | **Arterialized NEFA** | **Arterialized 1,25(OH)_2_D_3_** |
| --- | --- | --- | --- |
| 1.25(OH)_2_D_3_ flux during ISO-stimulation | 0.402 (P=0.154) | 0.341 (P=0.233) | 0.240 (P=0.409) |

Data are presented as correlation coefficient (P value).
